# The First Sign of Recurrent Angioimmunoblastic T-Cell Lymphoma: A Cutaneous Presentation

**DOI:** 10.7759/cureus.44805

**Published:** 2023-09-06

**Authors:** Guy Charest, Michael McBride, Amanda K Thomas, Mitch Manway, David J DiCaudo

**Affiliations:** 1 Dermatology, HonorHealth Dermatology Residency Program, Scottsdale, USA; 2 Dermatology, Creighton University School of Medicine, Phoenix, USA; 3 Dermatology, Mayo Clinic Dermatology, Scottsdale, USA

**Keywords:** ptcl, aitl, epstein- barr virus, peripheral t cell lymphoma, angioimmunoblastic t cell lymphoma

## Abstract

Angioimmunoblastic T-cell lymphoma (AITL) is a subtype of peripheral T-cell lymphoma with a nonspecific clinical presentation. Cutaneous manifestations of AITL are variable and include morbilliform eruptions, urticaria, papulonodules, and erythroderma.

We present the case of a 74-year-old male with a medical history of AITL presenting with diffuse erythematous macules and papules coalescing into patches and plaques on the trunk and bilateral upper extremities. Histopathology demonstrated a mild perivascular lymphocytic infiltrate in the dermis. By immunohistochemistry, the lymphocytic infiltrate was strongly positive for programmed cell death protein 1 (PD-1) (CD279) as well as cluster of differentiation 3 (CD3), CD5, and (focally) B-cell lymphoma-6 (BCL-6). Many cells within the infiltrate were positive for Epstein-Barr virus (EBV) by in situ hybridization. Additionally, a bone marrow biopsy demonstrated an atypical lymphoid infiltrate with T-cell predominance, many EBV-positive cells, and clonal T-cell receptor (TCR) beta gene rearrangement. Based on these histopathological findings, a diagnosis of recurrent AITL with cutaneous involvement was made. This case is a rare example of skin findings presenting as a first sign of recurrent AITL.

## Introduction

Angioimmunoblastic T-cell lymphoma (AITL) is an aggressive subtype of peripheral T-cell lymphoma (PTCL) of follicular T-helper cell origin [1]. It comprises 15%-20% of PTCLs and up to 2% of all non-Hodgkin lymphomas [2]. Angioimmunoblastic T-cell lymphoma usually presents in middle-aged and elderly adults at an advanced stage with fever, weight loss, night sweats, generalized lymphadenopathy, and hepatosplenomegaly. Cutaneous manifestations, including macules, papules, plaques, or nodules, are present in up to half of patients with AITL [3]. Given its variable presentation, AITL is difficult to diagnose, leading to poor outcomes and a median survival of less than three years [4, 5]. We present a case of the first sign of recurrent AITL, presenting as diffuse erythematous macules and papules coalescing into patches and plaques on the trunk and bilateral upper extremities.

## Case presentation

A 74-year-old male with a medical history of AITL presented with a rash on the trunk and bilateral upper extremities that had been present for six weeks. One year prior, he received chemotherapy for his AITL, which was complicated by leukocytic vasculitis with microthrombi affecting the distal fingertips. He was hospitalized with fever, chills, generalized weakness, encephalopathy, and a cough. Treatment with broad-spectrum antibiotics, antivirals, and antifungals was initiated before dermatology was consulted for evaluation.

The physical exam was remarkable for diffuse erythematous macules and papules coalescing into patches and plaques on the trunk and bilateral upper extremities, with sparing of the oral mucosa and lower extremities. The patient denied itching, burning, scaling, blistering, and oral involvement, as well as any history of atopic dermatitis, psoriasis, HIV, coccidioidomycosis, new medications, recent travel, animal exposure, or sick contacts. A full infectious workup was negative, and MRI and CT of the head and CT of the chest, abdomen, and pelvis showed no significant findings.

**Figure 1:**
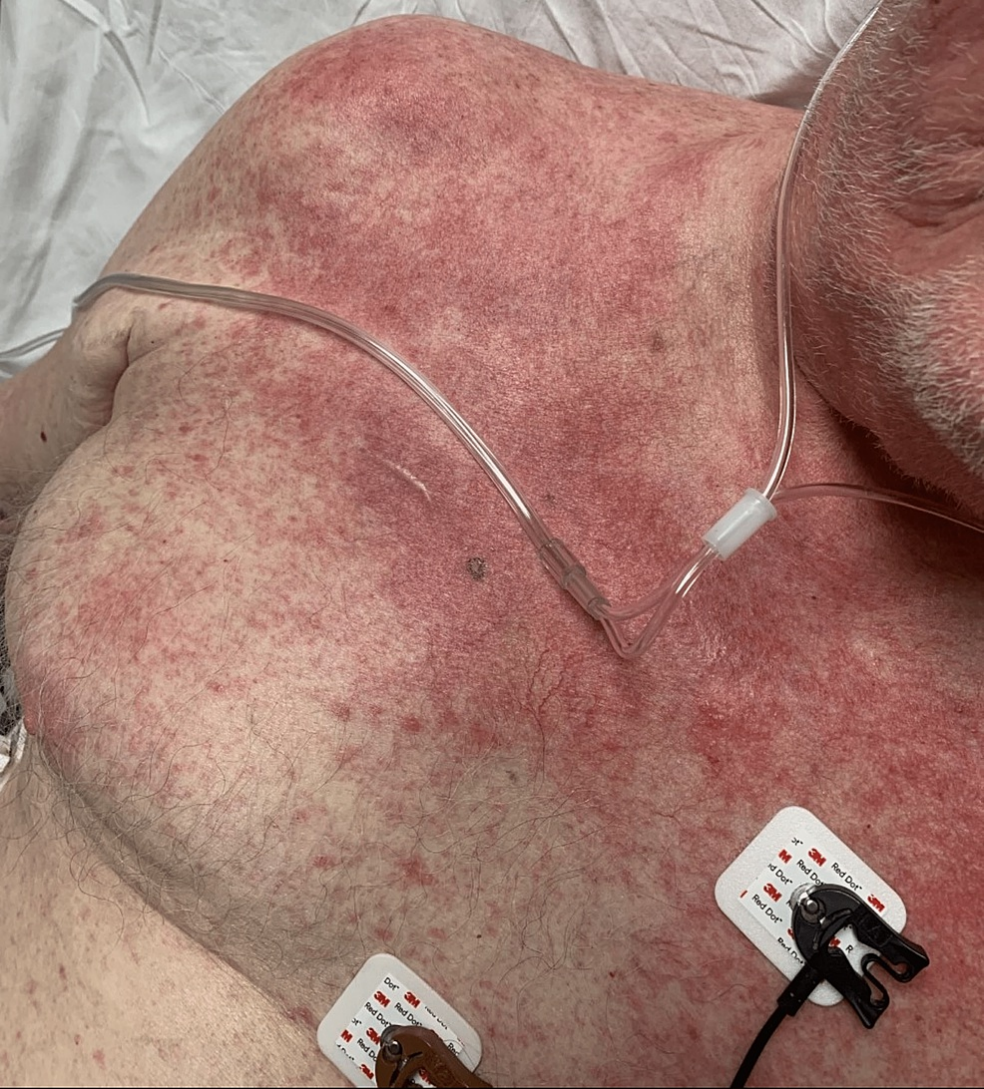
Diffuse erythematous macules and papules coalescing into patches and plaques on the trunk

**Figure 2:**
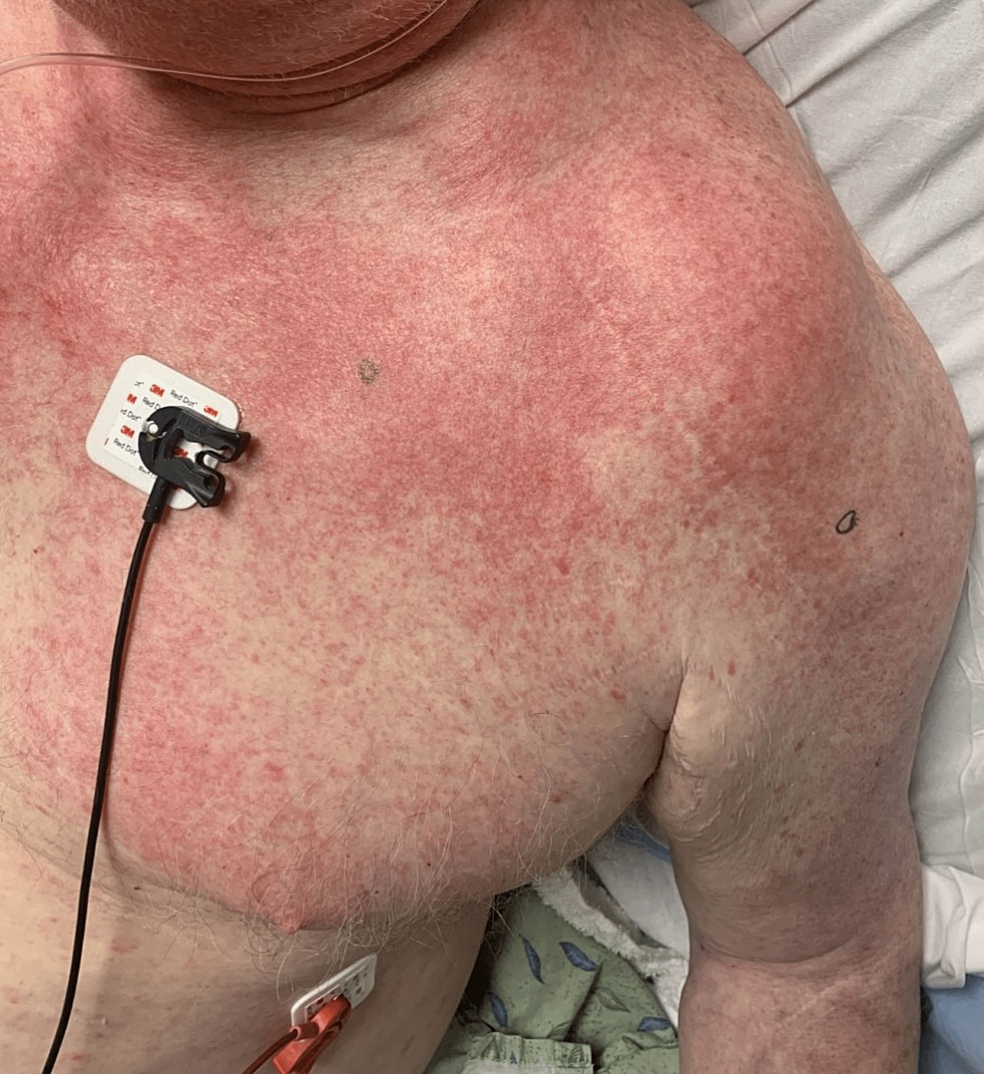
Diffuse erythematous macules and papules coalescing into patches and plaques of the left upper extremity

Given the physical exam findings, the differential diagnosis at the time of presentation included viral exanthem, drug reaction, and recurrent AITL. Multiple punch biopsies were performed (Figures 1-2).

**Figure 3:**
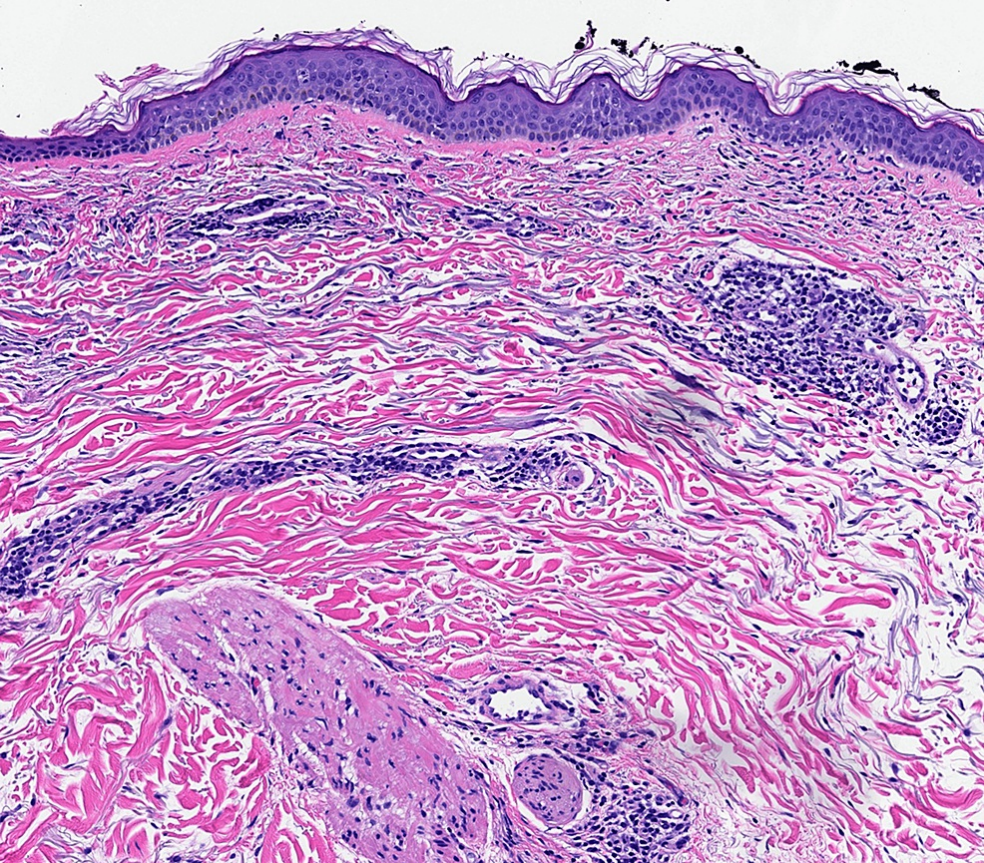
H&E x200: Mild perivascular lymphocytic infiltrate in the dermis

Histopathology demonstrated a mild perivascular lymphocytic infiltrate in the dermis (Figure 3).

**Figure 4:**
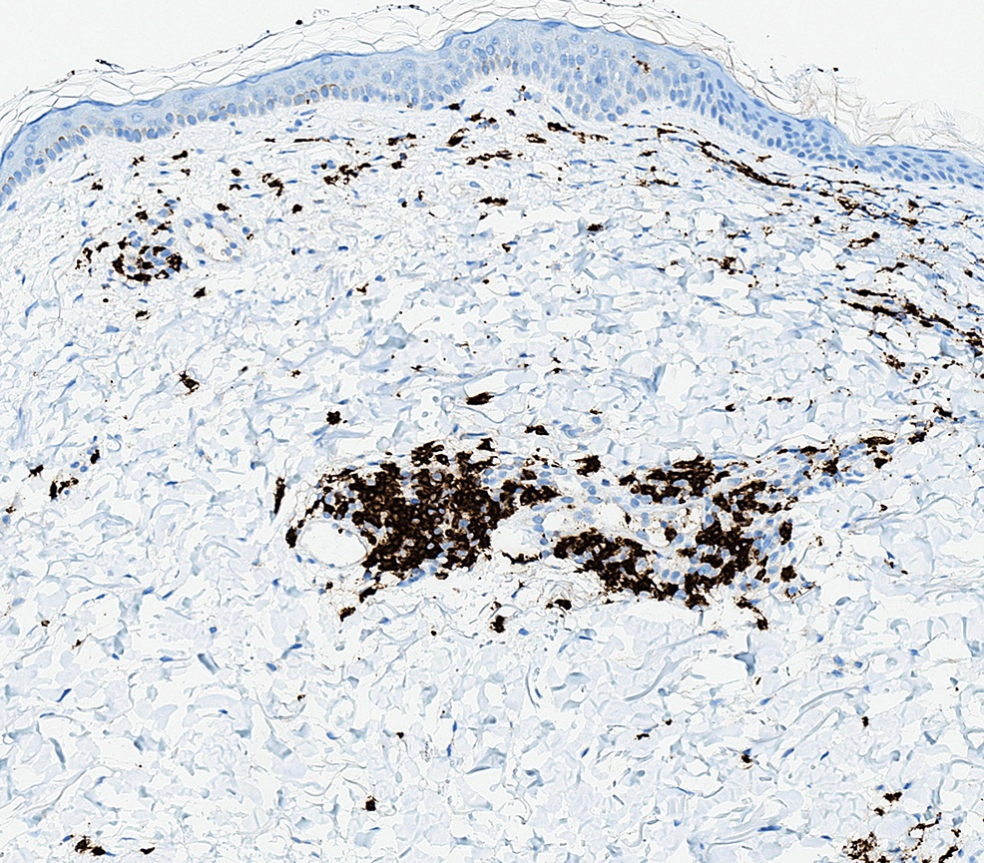
Lymphocytic infiltrate was strongly positive for PD-1 (CD279) on IHC x200 PD-1:  programmed cell death protein 1; IHC: immunohistochemistry

Immunohistochemistry demonstrated that the lymphocytic infiltrate was strongly positive for programmed cell death protein 1 (PD-1) (CD279) (Figure 4), as well as cluster of differentiation 3 (CD3), CD5, and (focally) B-cell lymphoma-6 (BCL-6).

**Figure 5:**
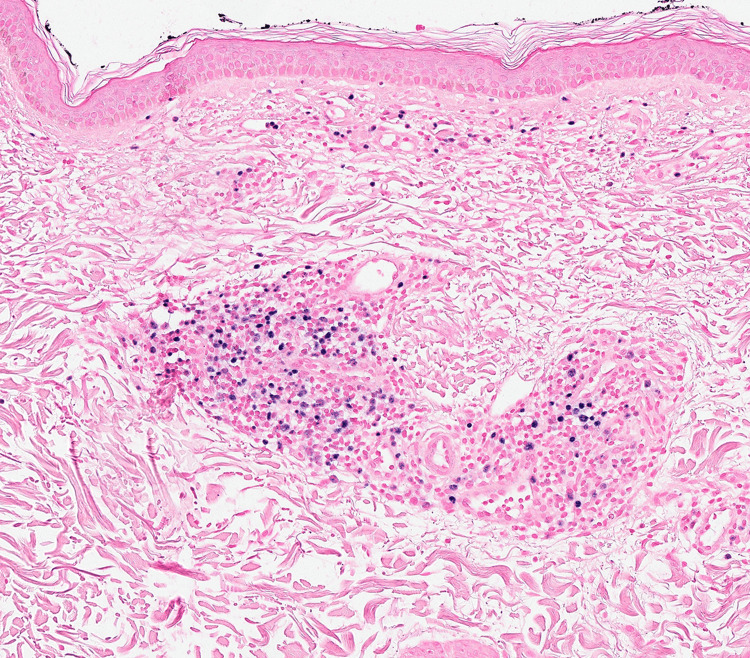
Many cells within the infiltrate were positive for EBV by in situ hybridization x200 EBV: Epstein–Barr virus

In situ hybridization revealed that many cells within the infiltrate were positive for Epstein-Barr virus (EBV) (Figure 5).

A bone marrow biopsy also revealed an atypical lymphoid infiltrate with T-cell predominance, many EBV-positive cells, and clonal T-cell receptor (TCR) beta gene rearrangement. A diagnosis of recurrent AITL with cutaneous involvement was made in light of these histopathological findings. High-dose prednisone was initiated due to previous intolerance to chemotherapy. He was discharged to a rehabilitation center and followed closely with oncology, ultimately passing away four months after discharge.

## Discussion

Angioimmunoblastic T-cell lymphoma presents with nonspecific clinical manifestations, making diagnosis a challenge. Patients can present with fever, weight loss, night sweats, generalized lymphadenopathy, and hepatosplenomegaly [3]. Immune dysregulation, leading to immunodeficiency and autoimmune complications, is also common [1]. Cutaneous findings are present in up to half of patients and include morbilliform eruptions, petechiae, urticaria, purpura, papulonodules, and erythroderma [3]. Pruritis is also a common finding in patients with AITL [6]. Given the heterogeneous presentation, the diagnosis of AITL based on cutaneous findings poses a challenge. This case is an example of skin findings presenting as a first sign of recurrent AITL. Biopsy and immunohistochemistry are often helpful in establishing the diagnosis, with most skin biopsies exhibiting perivascular infiltrate, vascular hyperplasia, or vasculitis [6]. Angioimmunoblastic T-cell lymphomas are of follicular T-helper cell origin and common markers include CD10, BCL6, PD-1/CD279, inducible costimulator (ICOS), C-X-C chemokine receptor type 5 (CXC-R5), chemokine (C-X-C motif) ligand 13 (CXCL13), and CD154, with at least two required to make a diagnosis [2]. Epstein-Barr virus-positive B-cells, admixed with neoplastic T-cells, are also a characteristic finding [1, 2].

Peripheral T-cell lymphoma is a class of relatively rare malignancies with limited reports on treatment outcomes. The most common chemotherapy regimen used in the treatment of AITL is cyclophosphamide, daunorubicin, vincristine, and prednisone (CHOP) [5]. Treatment with corticosteroids has also been shown to be effective for short remissions [7].

Angioimmunoblastic T-cell lymphoma usually presents at an advanced stage, with one study reporting that 90% of patients present in stage III or IV. The diagnosis carries a poor prognosis, with a median survival of less than three years and an overall survival of five and a half months with relapsed or refractory disease [3, 5, 8].

## Conclusions

Angioimmunoblastic T-cell lymphoma has a diverse clinical presentation, making diagnosis challenging. We present a case of recurrent AITL with diffuse erythematous macules and papules coalescing into patches and plaques on the trunk and bilateral upper extremities as the first sign of recurrence. Angioimmunoblastic T-cell lymphoma is usually diagnosed at an advanced stage given its variable presentation; thus, a high clinical suspicion for recurrent AITL should be considered in patients with new cutaneous findings. One study reported that 90% of patients present in stages III or IV. The diagnosis carries a poor prognosis, with an overall survival of only five and a half months and a median survival of less than three years with relapsed or refractory disease.

## References

[1] Xie Y, Jaffe ES (2021). How I diagnose angioimmunoblastic T-cell lymphoma. Am J Clin Pathol.

[2] Advani RH, Skrypets T, Civallero M (2021). Outcomes and prognostic factors in angioimmunoblastic T-cell lymphoma: final report from the International T-cell Project. Blood.

[3] Winfield HL, Smoller BR (2018). Other lymphoproliferative and myeloproliferative diseases. Dermatology, Third Edition.

[4] Elston D, Ferringer T, Ko C, Peckham S, High WA, DiCaudo DJ (2018). Cutaneous T-cell lymphoma, NK-cell lymphoma and myeloid leukemia. Dermatopathology, Third Edition.

[5] Rangoonwala HI, Cascella M (2023). Peripheral T-Cell Lymphoma. https://www.ncbi.nlm.nih.gov/books/NBK562301/.

[6] Balaraman B, Conley JA, Sheinbein DM (2011). Evaluation of cutaneous angioimmunoblastic T-cell lymphoma. J Am Acad Dermatol.

[7] Alizadeh AA, Advani RH (2008). Evaluation and management of angioimmunoblastic T-cell lymphoma: a review of current approaches and future strategies. Clin Adv Hematol Oncol.

[8] Siegert W, Nerl C, Agthe A (1995). Angioimmunoblastic lymphadenopathy (AILD)-type T-cell lymphoma: prognostic impact of clinical observations and laboratory findings at presentation. The Kiel Lymphoma Study Group. Ann Oncol.

